# Lessons learned from applying adaptation pathways in flood risk management and challenges for the further development of this approach

**DOI:** 10.1007/s11027-017-9773-9

**Published:** 2017-12-22

**Authors:** Pieter Bloemen, Tim Reeder, Chris Zevenbergen, Jeroen Rijke, Ashley Kingsborough

**Affiliations:** 1 Staff Delta Programme Commissioner, The Hague, Netherlands; 2 IHE Delft Institute for Water Education, Delft, The Netherlands; 3 Southampton University, Southampton, UK; 4 HAN University of Applied Sciences, Arnhem, The Netherlands; 5 University of Oxford, Oxford, UK

**Keywords:** Adaptation pathways, Climate change, Flood risk management, Lessons learned, Uncertainty

## Abstract

Worldwide, an increase in flood damage is observed. Governments are looking for effective ways to protect lives, buildings, and infrastructure. At the same time, a large investment gap seems to exist—a big difference between what should necessarily be done to curb the increase in damage and what is actually being done. Decision-makers involved in climate adaptation are facing fundamental (so-called deep) uncertainties. In the course of time, the scientific community has developed a wide range of different approaches for dealing with these uncertainties. One of these approaches, adaptation pathways, is gaining traction as a way of framing and informing climate adaptation. But research shows that “very little work has been done to evaluate the current use of adaptation pathways and its utility to practitioners and decision makers” (Lin et al. 2017, p. 387). With this paper, the authors, as action researchers and practitioners involved in two of the world’s largest real-life applications of this approach in flood risk management, aim to contribute to filling in that gap. Analysis of the experience in the United Kingdom and the Netherlands in long-term planning in flood risk management shows that the adaptation pathways approach is effective in keeping decision processes going forward, to the final approval of a long-term plan, and helps increase awareness about uncertainties. It contributes to political support for keeping long-term options open and motivates decision-makers to modify their plans to better accommodate future conditions. When it comes to implementing the plans, there are still some major challenges, yet to be addressed, amongst others: the timely detection of tipping points in situations with large natural variability, the inclusion of measures that prepare for a switch to transformational strategies, and the retention of commitment of regional and local authorities, non government organizations, and the private sector, to climate adaptation as national policies move from blueprint planning to adaptive plans. In delivering this feedback, the authors hope to motivate the scientific community to take on these challenges.

## Introduction

It is now clear that climate change and socio-economic growth lead to a growing need for considering the robustness and flexibility of structures, systems, and plans (Milly et al. 2008; Hallegatte 2009; Hallegatte et al. 2012; Kalra et al. 2014; Walker et al. 2013; Petersen and Bloemen 2014).

The editorial of the Global Environmental Change special edition that looked at the growing body of literature on adaptation to climate change (Maru and Smith 2014, p.322) concluded

“These efforts have been important in the progression of this area of research, improving understanding of climate-change adaptation related problems, and informing adaptation planning. However, it is widely reported that these efforts have not yet had a large impact on the implementation of adaptation plans (reviewed in Wise et al. 2014).”

An important aspect of climate change adaptation is managing the deep uncertainties related to future changes in climatic and social economic conditions in the development of strategies and the design of measures (Walker et al. 2001; Werners et al. 2015; Bloemen 2015).

As summarized by Kwakkel et al. (2016),

“Deep uncertainty means that the various parties to a decision do not know or cannot agree on the system and its boundaries; the outcomes of interest and their relative importance; the prior probability distribution for uncertain inputs to the system (Lempert et al. 2003; Walker et al. 2013); or decisions are made over time in dynamic interaction with the system and cannot be considered independently (Haasnoot et al. 2013; Hallegatte et al. 2012).”

The notion of deep uncertainty has spurred the development of adaptive policymaking. A generic trade of adaptive policymaking is that, in contrast to static policies, it results in contingency plans and specified conditions under which the policy should be reconsidered (Walker et al. 2001). The ability to change policy practices based on new experience and insights is an important aspect of adaptive management (Pahl-Wostl 2007). Klijn et al. (2015) state that adaptive management should rely on a sound ex-ante policy analysis which encompasses a future outlook, establishing whether policy transition is required, an assessment of alternative flood risk management strategies, and their planning in anticipation without running the risk of regret of doing too little too late or too much too early.

Climate change adaptation is a rapidly expanding field of expertise. While the practical experience is gradually growing, the field of expertise as a whole is still dominated by science focusing on, amongst others, the development of rational approaches for dealing with uncertainty (Wise et al. 2014). Policymakers, politicians, and other decision-makers are increasingly interested in information on the practical applicability of these approaches and scientists do try to meet this demand (Bradfield et al. 2016; Ben-Haim 2015; Convery and Wagner 2016; Derbyshire and Wright 2017; Dewulf and Termeer 2015; Kwakkel et al. 2016; Lempert et al. 2016; Lyons and Davidson 2016; Maier et al. 2016; Whaley and Weatherhead 2016).

One of the approaches that has been developed to respond to deep uncertainty is the adaptation pathways or route-map approach (Reeder and Ranger 2011; Haasnoot et al. 2013; Wise et al. 2014). A route map or adaptation pathway map illustrates a series of interrelated adaptation pathways. Adaptation pathways show what options make sense under what conditions and indicate in what timeframes these conditions can be expected under certain climate scenarios (Kwadijk et al. 2010).

“It provides decision makers a way to acknowledge the inter-temporal complexities and uncertainties associated with the novel dynamics of climate change and a mechanism to manage these challenges in the local context.” (Lin et al. 2017, p. 384) “Because of the recognized complexity of decision-making under climate change uncertainty, applications of adaptation pathways have been rapidly growing around the world in the hope and expectation that it will assist in overcoming constraints and barriers to disaster mitigation and climate adaptation.” (p. 386) “However, very little work has been done to evaluate to current use of adaptation pathways and its utility to practitioners and decision makers.” (p. 387) This paper aims to fill in exactly that gap.

The experience with two of world’s largest real-life applications of adaptation pathways in the field of flood risk management is analyzed in order to distill lessons learned and formulate challenges for the further development of this approach. As Klein et al. (2017) conclude in their report, “Advancing climate adaptation practices and solutions - Emerging research priorities”: “One clear opportunity for improvement is the nexus between adaptation researchers, and practitioners focused on adaptation action. There is a need to better understand how knowledge is transmitted and diffused, and the role that communication plays in policy learning. It is also increasingly recognized that effective research-practitioner engagement operates in both directions and requires true collaboration and mutual learning. By drawing on insights from a variety of perspectives and contexts, including practitioners, researchers can produce higher- quality work and build relationships that will facilitate research uptake.” (p. 13).

The further development of the adaptation pathways approach needs, just like other complex policymaking concepts, an effective science-policy debate, by inviting scientists in the policy world and vice versa. This paper is written by action researchers and practitioners and aims to distill lessons learned from applying the adaptation pathway approach in practice in the field of flood risk management.

## Adaptation pathways

Adaptation can be seen as a dynamic, long-term transitional process involving repeated decisions that can be structured using the adaptation pathway approach. Maru and Smith (2014) conclude that the development of the concept of adaptation pathways is informed by at least four strands of research, each inspired by different framing of adaptation pathways. The first involves the framing of adaptation pathways in the context of sustainability and development. A second strand responds to deep uncertainties around long-term levels of change in adaptation decision-making. The third strand concerns the depth of change required for robust and equitable adaptation, as observed from studies and theories of change. The fourth and last strand is the need for a detailed causal understanding of how climate change and extreme events lead to impacts and possible adaptation responses.

Adaptation pathways provide “an analytical approach for exploring and sequencing a set of possible actions based on alternative external developments over time.” (Haasnoot et al. 2012, p. 485). Following the definition of Barnett et al. (2014, p. 1103), an “adaptation pathway is a decision strategy that entails a vision for the entity exposed to climate risks, to be met through a sequence of manageable steps over time, each of which is triggered by changing environmental or social conditions.” Main features of the approach are that it takes into account multiple possible futures and that it foresees adjustments of plans as conditions change. It therefore requires a focused monitoring program that is transparently linked to decision-making processes at different regional and temporal scales. The Dynamic Adaptive Pathways Planning (DAPP) approach (Haasnoot et al. 2013) has adaptation pathways at its core. DAPP has been used increasingly for implementing climate-resilient pathways for water management.

In the case of flood risk management, the process of developing an adaptation pathway typically starts with evaluating the current level of flood risk and the standards of protection and mapping future sensitivities to climate change, like (upper bounds of) sea level rise. Known or estimated key thresholds between the present and these upper bounds are assessed in terms of vulnerability to impacts. In the case of a storm surge barrier, a key threshold might be the sea level rise at which the current system will fall below the target protection level. Subsequently, feasible adaptation response options for coping with these thresholds are identified. Interactions with other issues such as development pressures are checked. Route maps of response options that will tackle the thresholds are assembled. Costs and benefits and other criteria (like environmental impacts) of each route are compared under the most likely rate of change in extreme water level. That information is used to recommend the preferred route under the most likely rate of change. Key variables are identified that should be monitored to assess if a switch of route will be needed in the future. In the implementation phase, significant deviations from the expected rate of sea level rise will inform decision-makers about the necessity to delay or accelerate the program.

Even in the adaptation pathways approach, it is possible that actions that seem evident now will turn out to be maladaptative as the actual risk situation reveals itself. But by explicitly taking into consideration a bandwidth of possible futures and by identifying long-term options and guaranteeing as much as possible that they are kept open, the risk of maladaptation is reduced.

Wise et al. (2014) warn for a too narrow framing of adaptation pathways. They observe a focus on proximate causes and incremental actions and a shortage of more systemic or transformative actions. They attribute this partly to the way the issue of adaptation is framed. Decision-oriented approaches presuppose governments able and willing to effectively address clearly defined unambiguous goals. A broader conceptualization of adaptation pathways inspired by the sustainability domain opens up the search for adequate responses. Aspects that thereby come into view are, e.g., path dependency, interactions between adaptation plans, and situations where values, interests, and institutions constrain societal responses to changing conditions.

For example, countries often require a certain cost-benefit ratio to be achieved in order for the (planned) investment adaptation pathways to be considered worthwhile. In theory, all schemes with a cost benefit of greater than 1 should be funded. But in eras of constraints in available investment finance, high ratios can be part of the need to ration the amount of available funding when allocating it to schemes. For instance, the ambition of the Environment Agency of the UK Department for Environment, Food and Rural Affairs for the overall flood investment program was to realize benefit-cost ratios approaching 8:1. Also in the Netherlands, benefit-cost ratios of large-scale public investments are preferably 1 or higher. Political considerations can result in decisions to realize investment projects with lower benefit-cost ratios. The Dutch Central Planning Bureau recently finished their research on the role of cost-benefit analysis and cost-effectiveness studies in decisions on large-scale investments for improving safety against flooding in the last century. The results show that most often, the political decision to realize a certain goal, like the decision to improve safety from flooding by the sea by means of shortening the coastline in the southwest of the Netherlands in 1954, or to improve safety from flooding by the rivers by means of enlarging the room for the rivers in 2005, is taken on political grounds. After that, decisions about how to realize these goals are based on a cost effectiveness study (Bos and Zwaneveld 2017).

Adaptation pathways were first applied in practice in developing the Thames Estuary 2100 Plan (Environment Agency UK 2012a; Ranger et al. 2013; Reeder and Ranger 2011) and in the Dutch Delta Programme on water safety and freshwater supply (Haasnoot et al. 2012; Morselt 2013; Rijke 2014). It has also been considered in, amongst others, water resource planning for London (Kingsborough et al. 2016); coastal planning in England; strategic regional planning on the Eyre Peninsula, Australia (Siebentritt et al. 2014); coastal planning in Lakes Entrance, Australia (Barnett et al. 2014); urban adaptation in New York to hurricane and storm surge risk (Rosenzweig and Solecki 2014); and flood risk management in the Hutt River, New Zealand (Lawrence et al. 2013). Recently, adaptation pathways have been constructed for heat-risk management in London (Kingsborough et al. 2017). Although for each of these cases, it has been described how the approach could in theory lead to more informed decision-making in the face of deep uncertainty; lessons about its practical applicability have not yet found their way back to the scientific debate.

## Methodology

The authors of this paper, action researchers actively involved in the Thames Estuary 2100 Programme and the Dutch Delta Programme, and practitioners with a central role in the application of the adaptation pathway approach in these programs, analyzed the experiences with the real life application of the adaptation pathway approach. In this paper, the authors reflect on the process and end results of the phase of strategy development. A systematic approach has been followed to collecting, compiling, and analyzing experiences with adaptation pathways approach (APW) to identify lessons learned and best practices.

The main source for deriving the lessons learned from these experiences is the discussions between the practitioners and the action researchers. In addition, use is made (in the case of the Delta Programme) of four surveys and evaluations that were done in the period 2013–2016. The first one is a large-scale survey of the Delta Programme in 2013 by the Erasmus University Rotterdam (Verkerk et al. 2014). The 1481 visitors of the Delta Congress 2013 were invited to contribute to an extensive survey (100 questions addressing 12 issues). Six hundred forty-five people filled in the questionnaire (43%). The second source is a study on the governance of the Delta Programme after the publication of the Delta Programme 2015 (van Buuren and Teisman 2014). A total of 13 representatives of national ministries, 23 representatives of regional steering groups responsible for the regional sub-programs, and 4 representatives of branch organizations of regional and local authorities were interviewed and 5 regular governmental meetings were used to exchange thoughts on the functioning and results of the Delta Programme. Source 3 is an independent review focused on the use and added value of Adaptive Delta Management (ADM) and the adaptation pathways approach in the Dutch Delta Programme (Rijke 2014). The sub-programs of the Delta Programme were interviewed and asked to contribute to an online survey. In addition, observations were made during meetings of the ADM core team as they prepared and evaluated the ADM training workshops for the regional teams responsible for constructing the adaptation pathways. The fourth source is a formal legislative ex-post evaluation of the Delta Act, executed in 2016 (Algemene Bestuursdienst TOP Consult 2016). In the context of that evaluation, 15 top key players were interviewed (deputies of provinces, mayors of cities, chairs of advisory committees, top authorities of waterboards, the minister responsible for water management, etcetera) and three thematic roundtables were organized with each on average 10 specialists in the fields of flood risk management, freshwater availability, and spatial planning. Input for the formal evaluation was also generated from discussions with the managers of the regional and thematic sub-programs of the Delta Programme and with 16 non government organizations (NGOs) involved in the same fields as the Delta Programme.

In the case of TE2100, additional sources of review have included a survey of key stakeholders carried out by Kingsborough (author) in connection with his work on piloting adaptation pathway methodologies for heat, water resources, and surface water flood risk in London, United Kingdom linked to the Greater London Authority (GLA) and the London Climate Change Partnership (LCCP). An initial review of project reports and stakeholder interviews was undertaken to understand how TE2100 incorporated adaptation monitoring and evaluation (M&E). Follow-up interviews were completed to identify which stakeholders are responsible for monitoring which indicators, who uses the collected information, and for what purpose. TE2100’s adaptation M&E approach was reviewed to learn more about the barriers and enablers of adaptation M&E (Kingsborough 2016; PhD thesis). Another key source was the TE2100 5 Year Monitoring Review, which provided detailed information and experience of monitoring and reviewing 10 key indicators. This was particularly useful in terms of highlighting the need for picking up changes in indicator trend from natural variability and points the way forward to useful research. It is one of, if not, the first comprehensive monitoring reviews of a major strategy based on adaptation^1^pathways. The results of the discussions between stakeholders and action researchers were combined with the results of the surveys and evaluations to formulate the experiences of applying adaptation pathways (see Section 5).

To avoid biased perceptions towards the authors’ own work, the respective experiences were discussed in a number of workshops during national and international conferences held in the period 2011–2015. Experiences in the Netherlands have been discussed with both scientists and practitioners in, amongst others, the National Delta Conference in 2011 (Amsterdam) and the European Conference on Climate Adaptation in 2013 (Hamburg) and 2015 (Kopenhagen). The United Kingdom (UK) experiences were discussed in, amongst others, the Sea level Rise Conference 2010 (Corpus Christi, Texas), the Sea level Rise Conference 2012 (Wellington, NZ); the Mekong Technical Workshop 2012 (Khon Kaen, Thailand), Water, a resource for the metropolis (2013) Paris; and the CIWEM National Conference (2014) London. Both cases were discussed in the international conference Deltas in Times of Climate Change in 2014 (Rotterdam) and the Decision Making Under Uncertainty working sessions in 2014 (Santa Monica) and 2015 (Delft). The insights gained in these conferences and workshops from presentations and discussions with scientists and practitioners were fed into the development of the ADM approach during meetings of the ADM core group.

**Fig. 1:**
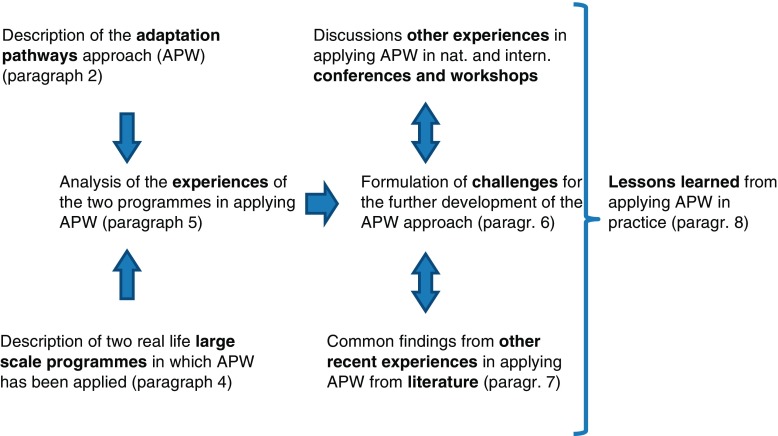
Process flow of this research

The results from these discussions formed the basis for formulating challenges for the further development of the approach (Section 6). The results have been compared with experiences from application of APW in programs from other domains, which have been documented in the scientific literature. After collecting, compiling, and analyzing the experiences with APW, lessons learned and best practice have been identified in Section 8. Figure 1 summarizes the process flow of the research.

## Two large water management programs in the UK and the Netherlands

### UK plans on flood safety and resource planning

In the UK, the use of adaptation pathways was pioneered by the Thames Estuary 2100 (TE2100) project which produced a plan for managing tidal flood risk in the Thames estuary and London for the twenty-first century. TE2100 was one of the first major infrastructure projects to explicitly recognize and address the issue of the deep uncertainty in climate projections throughout the planning process (Ranger et al. 2013). The plan as a result has the uncertainties surrounding climate change impacts at its core.

A critical component in the development of the TE2100 Plan was a risk assessment, which included identifying existing levels of vulnerability, collating and generating climate information, assessing the sensitivity of existing flood risk management approaches to future climate, identifying thresholds relevant to decision-making (e.g., values for mean sea level) that trigger the need to modify existing flood defenses), and identifying and appraising the effectiveness of potential adaptation actions (Ranger et al. 2013).

The development of the TE2100 Plan included appraising adaptation portfolios using multi-criteria assessment and prioritization of preferred pathways (Ranger et al. 2013). The TE2100 plan has a set of options based on adaptation pathways which can cope with increases in maximum water levels from those experienced at the start of the century through to a worst case of 2.7 m by 2100 (Environment Agency UK 2012a). The preferred pathway identified includes staged long-term modification of the Thames Barrier and the management of fluvial and pluvial flooding through local measures including making space for water, local flood defenses, building resilience measures, flood forecasting, and emergency planning (Environment Agency UK 2012a). The project also explored potential limits of adaptation,^2^ which are anticipated if sea level rise exceeds 5 m.

**Fig. 2:**
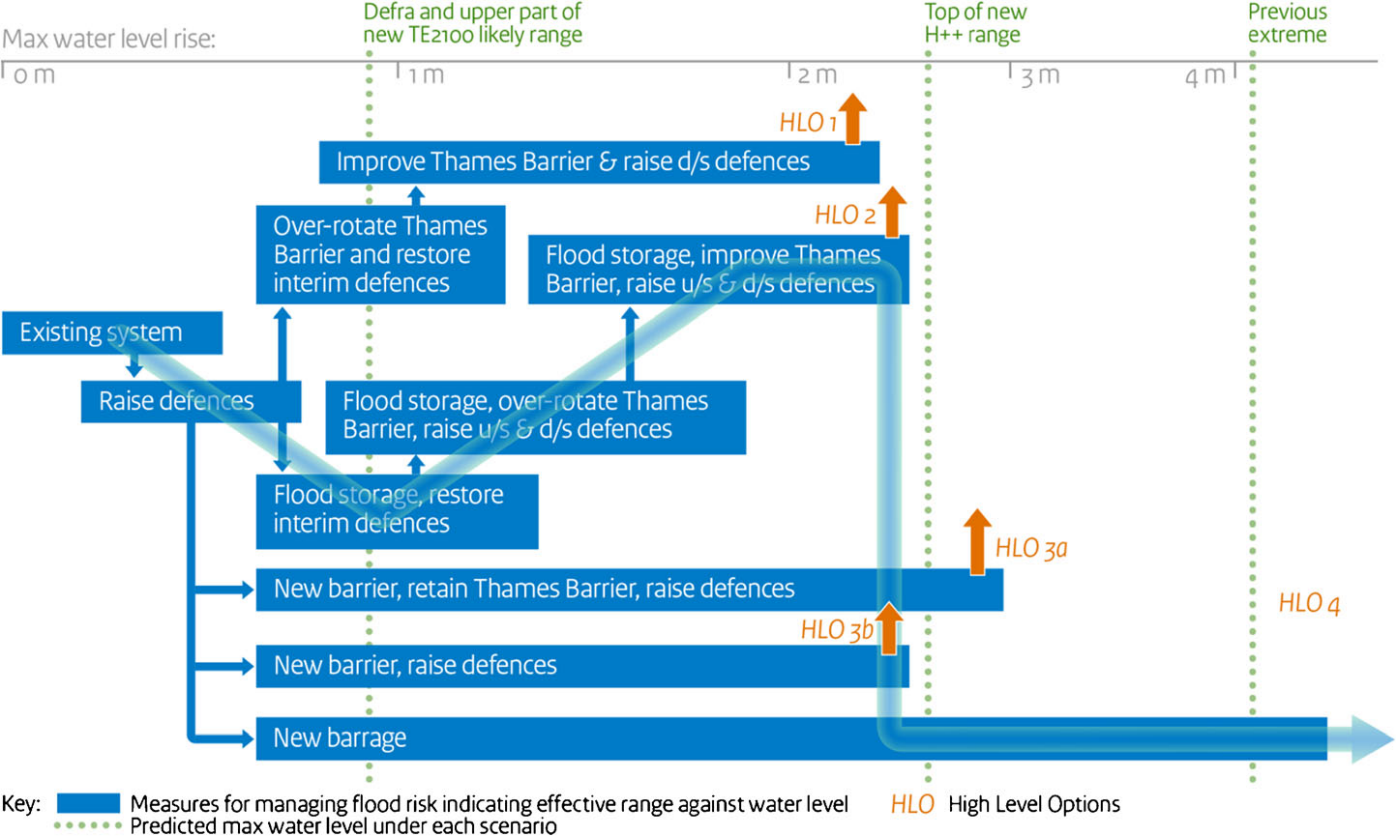
Adaptation pathway map for the Thames Estuary (Environment Agency UK 2012a)

Figure 2 shows how options can be combined to achieve a plan where investment in a barrage is delayed until it is needed. An “Option” is a number of portfolios which, when implemented in sequence, provide a complete flood risk management solution for the next 100 years. A “Portfolio” of responses is a number of responses which, when combined together, provide a complete flood risk management solution for a particular increase in sea level and/or fluvial flow. A “Response” is an individual flood risk management measure, for example, a barrier, a length of raised defense, or an emergency plan for a community (Environment Agency UK 2012b).

In the TE2100 Project, socio-economic scenarios were used to compare the effectiveness of differing adaptation pathways. The plan needed to consider not only growing hazards due to climate change and aging infrastructure, but also the rising economic value at risk and population at risk throughout the Estuary. The plan needed to meet a range of environmental and social objectives (Environment Agency UK 2012a) and value/cost benefit was a key part of the decision-making process (Penning-Rowsell et al. 2013). There was an extensive strategic environmental assessment (Environment Agency UK 2012b) and MCDA (Penning-Rowsell et al. 2013).

The plan has been approved by government and has set out a program of measures including a possible date for a new barrier (2070) assuming a 90-cm increase in water levels by 2100. The plan sets out how the timing of the measures may need to change depending on the monitoring of key indicators including sea level rise, river flow, and erosion rates. The outputs from the monitoring program will inform the scheduled review and re-appraisal of the TE2100 Plan every 10 years, with a mid-term monitoring review to be undertaken every 5 years. The first 10-year program of work has been approved with a £350 million budget. TE2100 sets a long-term strategic vision of how London can adapt and establishes the potential need for transformational change in the long term.

### The Dutch Delta Programme

In the Dutch Delta Programme, the national government, provinces, municipalities, and water boards work together on the improvement of flood risk management and on the reduction of vulnerability to water scarcity (Delta Programme Commissioner 2011; Van Alphen 2015). The program follows a proactive approach to flood prevention instead of preparing for flood disasters (Schultz van Haegen and Wieriks 2015). Reducing disaster risk is a cost-effective investment in preventing future losses (Zevenbergen et al. 2013; Sendai Framework for Disaster Risk Reduction 2015–2030).

In countries with stringent flood safety standards like the Netherlands, there are no indications that the risk decrease resulting from a further local reduction in the probability of a flooding is nullified by a risk increase due to higher consequences of a flooding resulting from an accelerated growth in population and investments.

Involving social organizations and the business community, the public authorities prepared the five so-called “Delta Decisions.” These over-arching decisions form the basis of the work that the Netherlands will perform over the next 35 years (with a planning horizon of 2100). The decisions concern new water safety standards, sustainable fresh water provision, climate-resilient design and construction of urban and rural areas across the Netherlands, and structuring choices for flood risk management and freshwater supply in the IJsselmeer region and the Rhine-Meuse delta. Six regional strategies were developed iteratively, consisting of goals, measures, and a tentative timeline. These regional strategies were developed in the regional sub-program of the Delta Programme, in which provinces, municipalities, and water boards work together, involving the scientific community, NGOs and the private sector.

Four so-called “Delta Scenarios” were developed to guide the process of formulating the Delta Decisions and constructing the regional strategies. These scenarios cover the two main uncertainties: climate change and socio-economic conditions. A rough indicative analysis of the reasons for investing in improving the protection system against flooding shows that approximately 40% can be assigned to climate change and land subsidence and 60% to a backlog in maintenance of the present protection system in combination with outdated flood safety standards. These standards were based on the size of the population and value of investments in the early 1960s of the last century. The new standards, which came into effect on January 1, 2017, take into consideration the “high end” of the four Delta Scenarios. For 2050, the date on which the new protection level has to be realized, they assume considerable climate change (average temperature up 2°, sea level rise of 35 cm, winter precipitation up 14%) and an increase in population (20 million people) and value of investments (ongoing economic growth of 2.5% a year).

**Fig. 3:**
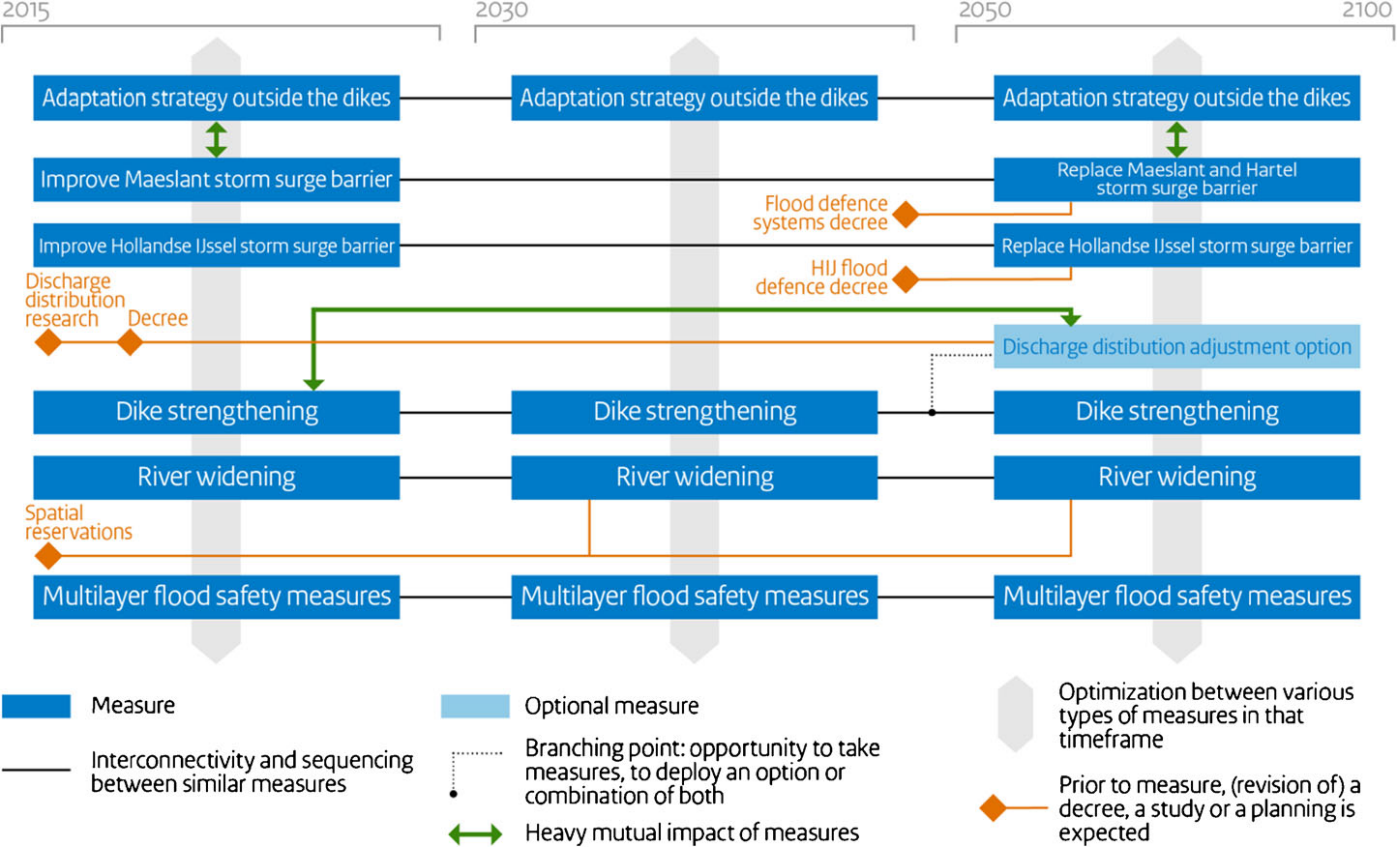
Adaptation pathway map for flood risk management in the Rotterdam area (Delta Programme Commissioner 2014)

The Delta Plan on flood risk management and the Delta Plan on freshwater, both financed from the Delta Fund, comprise the measures from the regional strategies. The “Delta Decisions,” regional strategies, and two Delta Plans formed the central elements of the proposal sent to parliament in September 2014. The proposal contains a total of 14 pathways, developed with a planning horizon until 2100. Figure 3 illustrates the pathway developed for the Rotterdam area. The proposal was accepted and the necessary budget until 2028, €17 billion, has been allocated (Delta Programme Commissioner 2014).

A “Signal Group Delta Programme” has been assigned the task to inform about developments and possible tipping points. Authoritative knowledge institutes in the field of water, spatial planning, and climate are represented in the Signal Group. The group reports on developments in three categories: “knowledge and innovation,” “climate change and socio-economic developments,” and “societal preferences.”

Figure 3 shows which measures (dark blue boxes) are considered in the three consecutive time periods: 2015–2030, 2030–2050, and 2050–2100. The horizontal trajectories visualize the strategies that will be implemented simultaneously. Light blue boxes indicate long-term options, like the modification of the discharge distribution among the Rhine branches. The orange diamonds indicate preparatory actions. Examples are spatial reservations, especially necessary for river widening, and research on the costs, benefits, and technical feasibility of modifying the discharge distribution among the Rhine branches. Green arrows highlight measures that heavily influence the effectiveness of other measures. If for instance the research on the possibilities of modifying the discharge distribution leads to a decree that this measure will be implemented in the future, then dike strengthening projects will need to include the effects of this modification in their planning and design.

## Experiences of applying the adaptation pathways approach in the two water management programs

Experience shows the adaptation pathways approach was effective in keeping decision processes going forward, to the final approval of a long-term plan. It helped increasing awareness about uncertainties, offered visualization of multiple alternatives, provided political support for keeping long-term options open, and motivated decision-makers to modify their plans to better accommodate future conditions. By making transparent how short-term decisions can be related to long-term tasks, it motivated and facilitated policymakers, politicians, and other decision-makers to incorporate uncertainty about future conditions in their decisions and plans.

Officially, earmarking land for future flood defenses in the TE2100 Plan and reservations for a the long-term option of the large-scale retention area in the upper Rhine region were ways of the government for assigning strategic value to parcels of land despite short- and medium-term development pressures. This puts constraints on the use of this land, which requires political willpower. The clear articulation of long-term pathways supported initiatives to secure the necessary long-term financing.

The experiences show that long-term pathways notably have added value if focussed at a strategic level of decision-making. A “free thinking space” should be fostered that does not restrict the consideration of actions that may not be politically or financially acceptable in the short term. Pathways approaches can be used to encourage the development and consideration of creative solutions.

Adopting a flexible approach to climate change based on adaptation pathways was critical in helping gain approval and buy in to the plan with key stakeholders—both in London and the wider South East and in the 6 regional sub-programs of the Dutch Delta Programme. The principles in terms of an adaptive approach to planning for the impacts of climate change have been built into the guidance for flood risk management undertaken by both the Environment and Agency and Lead Local Flood Authorities (Kuklicke and Demeritt 2016) and into the yearly Delta Programme report s that are submitted to parliament (Delta Programme Commissioner 2014, 2015, 2016).

Building consensus on the need for adaptive action and how to implement it is difficult (Barnett et al. 2014); however, experience shows that having a mock-up of adaptation pathway diagrams early in the development process is beneficial for communicating concepts and garnering stakeholder support.

There has as yet been no new example of implementation in the UK of an adaptive plan on the scale of the TE2100 Plan. However, the value of the TE2100 approach and its potential applicability is increasingly being recognized in the UK and abroad. The approach has been considered and used to prompt debate within several initiatives including the Coast Communities 2150 project which developed an indicative long-term adaptation plan for Newhaven on the South Coast of the UK. The London Climate Change Partnership has advocated for the use of pathways approaches and the London Assembly has called for the mayor to “formulate options for adaptation, grouped where appropriate into ‘pathways’ of linked adaptation” (London Environment Committee (LEC) 2015, p.27).

The large-scale survey in the Delta Programme in 2013 showed that 72% of all respondents agreed that the Delta Programme was successful in connecting short-term decisions with long-term objectives, one of the four goals of Adaptive Delta Management. The use of adaptation pathways, facilitating future switches between strategies, was judged positively by 47% of the respondents. A relatively high degree of 15% indicated that they felt they could not judge this aspect of Adaptive Delta Management. This issue will be addressed in future editions of the communication plan of the Delta Programme. The robustness of the preferred regional strategies was judged positively by 56%. Three roles of the top 5 most important roles assigned to the Delta Commissioner are directly related to Adaptive Delta Management and working with adaptation pathways: “Watch over the connection between short-term decisions and long-term objectives,” “Watch over the system approach,” and “Watch over the long term options; that they are effectively kept open.” The percentages of respondents that agreed on the high importance of these 3 roles are respectively 86, 80, and 80%.

After the publication of the Delta Programme 2015 report, the phase of strategy development formally ended, and the phase of the elaboration and implementation of the strategies started. Some of the responsibilities were transferred back to their original place in ministries and project execution organizations, and local and regional governments were challenged to coordinate the Delta Programme activities in their region. In the part of the survey that inventoried worries on that transfer of the responsibilities, 43% of the respondents indicated they were worried about maintaining, in this new situation, the adaptive capacity to deal with new and possibly unexpected conditions.

The study on the governance of the Delta Programme after the publication of the Delta Programme 2015 concluded that Adaptive Delta Management and thinking in adaptation pathways are important outcomes of the Delta Programme. They constitute a welcome transition in water management. It has emerged in the phase of strategy development and needs to be preserved in the face of elaboration and implementation. Three “core qualities” of the Delta Programme that need to be preserved in the next phase are “shared ownership,” “coherence,” and “adaptivity.” The necessity to maintain the adaptive character of the Delta Programme in the implementation phase was stressed by several organizations including research institutes (van Buuren and Teisman 2014).

An online survey was performed to gain insight into the experience in the Delta Programme of applying adaptation pathways in practice (Rijke 2014). It was executed on behalf of the staff of the Delta Programme Commissioner in order to find out which elements of the approach were considered most and least useful and which elements were most difficult, in order to further improve the approach and tailor assistance to the regional sub-programs of the Delta Programme where the strategy development was taking place. The survey addressed the people that actually worked on the formulation of these Delta Decisions and regional strategies, typically policymakers from different levels of government, consultants, and practice-oriented researchers. They assign the highest added value of applying adaptation pathways to the way it helps to incorporate long-term objectives (in flood safety, freshwater supply) in short-term decisions (in a broad range of sectors including water management, urban planning, nature, aging infrastructure, recreation, and shipping). The added value on this aspect rated 4.6 out of a maximum of 5 points. The contribution to increasing awareness of uncertainties was also rated highly (4.2), as was the positioning in a time frame of the measures (4.1), and promoting the development of strategies that can be adjusted to changing external conditions (4.1). Most difficult to apply in ADM were the determination of tipping points, the quantification of the added value of flexibility (real options analysis was considered too complex in a lot of cases), connecting with investment agendas of other organizations, and unraveling the interdependence of measures in different policy fields and different parts of the catchment area.

The formal legislative evaluation of the Delta Programme, executed in 2016, stresses the importance of maintaining adaptivity in the Delta Programme, but states that it is too early to judge its added value in the phase of elaboration and implementation. Maintaining adaptivity is seen as crucial. At the same time, it ranks in the top 5 of major challenges for the future. The evaluation commission concludes that it is too early to draw conclusions on the added value of adaptivity in the implementation phase of the Delta Programme. In the evaluation report, all parties involved in the Delta Programme are urged to acknowledge the importance of this specific aspect for the functioning of the Delta Programme.

## Challenges for the further development of the adaptation pathways approach

On the basis of an analysis of the practical experiences in the two water management programs, the results of discussing these experiences in national and international conferences, and the online survey on the applicability of the adaptation pathways approach in the Dutch Delta Programme, the following challenges were formulated for the further development of this approach.

### Determining tipping points in the absence of precise policy goals, for intrinsically flexible strategies and in situations with large natural variability

An implicit assumption of the adaptation pathway approach is that some physical parameter, be it climatic conditions influencing probabilities of a flood, or socio-economic developments influencing possible consequences of a flooding, changes gradually, thus slowly but surely forcing society to react and ultimately switch to a different strategy. This approach seems to work best in the case of gradual-trend-dominated developments like sea level rise, forcing a clear-cut decision on for instance the upgrade or replacement of a flood surge barrier.

Determining tipping points proved challenging in other contexts. Attempts to operationalize the adaptation pathways developed for freshwater availability in terms of defining when the next generation of measures should be implemented have temporarily been put on hold, as it has become clear that the policy objectives in that field were not precise enough to determine an approximate timing of the tipping points under the different climatologic and socio-economic scenarios (Knikpunten in zicht, Deltares 2017, in Dutch). The strategy chosen in the Delta Programme for the threats of flooding from the sea is “beach nourishment.” In this strategy, sand is supplied in the sea close to the coast, thus reducing wave erosion. This strategy is intrinsically flexible: every year, the volume of sand supplied can be enlarged or decreased depending on the rate of observed sea level rise. In that case, it is not possible—and not necessary—to determine tipping points.

The monitoring of the changes in the frequency of storms, droughts, and heat waves remains difficult, due to the lack of observations of extreme events, which are by definition rare. In the case of climate change-induced changes in peaks of river discharge, research combining monitoring data with model calculations shows that the natural variability in river discharge is so high that even when rapid (but not extreme) climate change is assumed, it can take 3 to 4 decades before the climate change signal can actually be distilled in a statistically sound way from monitoring data of river discharge (Diermanse et al. 2010; Klijn et al. 2012).

From a practical point of view, research is needed to find alternative approaches and/or parameters for distilling the climate change signals from river discharge measurements. This could be achieved through combining data-based detection of changes in observed events and exploration of possible future events through scenarios and modeling (Hall et al. 2014). Accordingly, Haasnoot et al. (2015) have identified a possible signaling role for decreasing summer river discharge as an indicator for changes in peak river discharge in the River Rhine. Dakos et al. (2015) point at the possibility of detecting early warning signals of a nearby tipping points by monitoring indicators of “critically slowing down.” Alternatively, large ensemble climate experiments (currently used for event attribution) may provide an alternate approach to better quantify the changing probability of extreme events (Kay et al. 2011; Pall et al. 2011). Such an approach could be used, for example, to probabilistically model the magnitude of the current 1 in 100-year heat wave event and compare this to the historic 1 in 100-year heat wave event. This could be updated every 10 years and thus becomes a useful tool in monitoring changing climate risk over extended timescales. Nevertheless, for a comprehensive approach for distilling climate signals from highly variable river discharge measurements is not yet readily available for policymaking purposes.

### Unraveling the relations between parallel strategies implemented simultaneously

The previously mentioned online survey that was performed to gain insight into the experience of applying adaptation pathways in practice (Rijke 2014) indicated that the most difficult to apply was the determination of tipping points (as described under point A) and unraveling the interdependence of measures in different fields and different parts of the catchment area.

In theory, adaptation pathways typically consist of several parallel trajectories and possibilities for switching from one trajectory to another when conditions indicate it might be wise to do so. In a given period, developments and measures follow one of these trajectories—depending on the context, actual conditions, and expectations about the future. The experience in the Dutch Delta Programme is that often, a *combination* of trajectories is chosen in the regional strategies. Parallel trajectories, reflecting different approaches and associated trajectories of action, are followed simultaneously.

In the case of flood safety, these parallel trajectories would, for example, be a multi-decade trajectory of regular dike reinforcement projects, a program consisting of a series of river bypass projects and a set of pilots for innovations in multi-level safety (i.e., prevention of flooding, protection during flooding, and preparedness for future flooding). In the domain of freshwater, one could see a parallel trajectory focusing on increasing the availability of freshwater being complemented by a trajectory focusing on the reduction of water use and another on improving water purification and a program for developing salt resistant crops (Delta Programme Commissioner 2014).

There are clear advantages to parallel systems (Jongejan et al. 2012). A strategy composed of several parallel trajectories contributes to the system’s resilience as it has more fallback options in case some of the trajectories do not perform the way they should. This is, for example, why the city of Dordrecht is interested in multi-layered safety: in case the primary defense system fails, the damage and casualties are reduced by adjusted spatial planning and up-to-date evacuation plans (Gersonius et al. 2015).

As different trajectories often address completely different actors and chances of successful implementation are uncertain, the interrelatedness of their outcomes is often given little attention. Determining the effectiveness of individual adaptation measures is already a complex matter (Klostermann et al. 2015); the simultaneous implementation of parallel trajectories further complicates the matter.

The efficiency of the complete strategy can be improved by investigating in an ex-ante evaluation how the different trajectories of action can both mutually strengthen each other, but also weaken each other, and adjust the strategy by recombining or eliminating the trajectories.

Practical experience in the Netherlands and the UK has shown that in the process of composing a strategy consisting of multiple parallel trajectories, it is useful to analyze ex-ante if the trajectories perform well under comparable or under contrasting conditions. In other words, it is recommended to analyze if the strategy as a whole will cover a wide spectrum of possible futures evenly, or if there is a skewness in the strategy for specific future conditions that should be compensated for.

### Maximizing broad societal commitment in situations of low predictability

Adaptation pathways make explicit what measures can be taken in the short term and sketch possible future measures. Decisions about these future measures can be taken in due time. The fact that final decisions about the actual implementation of these future measures are (as long as dramatic events do not happen) often not taken before physical conditions (climatic, socio-economic), justifying them are actually met—or can be predicted with relative certainty—implying that societal anticipation to these measures is hindered.

For instance, as long as the decision to increase freshwater availability is not taken, farmers arguably will hesitate to invest heavily in expansions of their business that increase the dependency on abundant availability of freshwater, because that availability will be harder to guarantee in most climate scenarios if large-scale interventions are not taken. So by postponing the final decision to execute the measure, “unwanted” anticipation is prevented. The described anticipation is “unwanted” because the expansion of the business would increase the dependency of abundant freshwater, where the original goal of the intervention was to promote resiliency of the agricultural sector by decreasing this dependency.

On the other side, taking the final decision not to execute the measure in the short term has the distinct advantage that more actors are challenged to prevent an increasing dependence of freshwater in other ways. For instance, companies can choose to invest in innovative purification methods or new salt-resistant crops.

Depending on the nature of the measure, on the costs and benefits for different actors of anticipating the measure, and on the direction the anticipation works in relation to the direction that was meant by the measure itself, the postponing of the final decision can constitute a net advantage or a net disadvantage.

These trade-offs should be taken in consideration in planning the moment for making the final decision about the actual implementation of a measure.

### Preparing the switch from incremental to transformational strategies

While in theory, the pathways approach is “neutral” to the choice of the type and order of measures, practice shows that the selected pathway or the preferred strategy often contains incremental measures in the short-term, firmer measures in the mid-term and (options for) system-changing interventions or transformational measures in the long term. The rationale behind it seems obvious: the longer the time-horizon, the larger the climatic challenges, thus the heavier the interventions.

Of course, the distinction between these categories is gradual and depends on the geographical and timescale that is considered. Using a geographical scale of several hundreds of kilometers and a timescale of several decades, concrete measures can be used to typify the three categories. Incremental measures are for instance series of local dike strengthening projects. Firm measures constitute a more robust approach, designed to meet upfront the more challenging of plausible futures. An example is the construction of regional bypasses in a river system to reduce the risk of flooding. Transformational measures drastically change the present system, preparing it to counter the most extreme situations. A typical example would be the construction of a new dam in an otherwise open estuary.

Incremental measures are “protective” in the sense that they can be considered as investments in a further gradual improvement of the resilience of the present system. Flipside is that this may increase the transfer costs to a new or significantly modified system. Increasing the resilience of the present system may also lead to an increase of sunk costs, further heightening the threshold for switching from an incremental strategy to a transformational strategy. Research on ancient societies has shown that sunk-cost effects can increase the vulnerability of a society (Scheffer et al. 2003; Janssen et al. 2003; Scheffer and Westley 2007). This is one of several psychological barriers that limit both climate change mitigation and adaptation (Gifford 2011).

Continuing on the path of incremental measures may enlarge path dependency. Useful information on this effect might be generated by comparing alternative adaptation pathways using a cost-benefit analysis that covers the complete period of the adaptation pathways (several decades, a century) and takes into consideration the issues of sunk costs and transfer costs. Specific attention is needed for dealing with the fact that discounting may blur the picture at the long-term end of the trajectory.

It is often stated that there are many plans for transformational measures, but that these measures are implemented only as a reaction to extreme events. Comparable to the TE 2100 Plan, the Delta Programme aims “to stay ahead of major floodings.” Due to climate change, transformational measures are inevitable in the long term. So at some point in time, the transition from incremental strategies to transformational strategies will have to be made. Though several authors have addressed the difficulty of making a *planned* shift to transformational strategies (Folke et al. 2010; de Haan et al. 2014; Kates et al. 2012; Lonsdale et al. 2015; Rijke et al. 2013), it has not been tackled yet adequately.

From this analysis, it also follows that middle-term investments in the resilience of the present system should be re-evaluated in the light of a possible future transition to a significantly modified system. Options are to adopt shorter depreciation periods, or to consider alternative measures that are specifically designed for relatively short periods.

A challenge for the adaptation pathways approach is to visualize (increases in) the path dependency of a strategy and (in) the transfer costs related to switching from one strategy to another (within an adaptation pathway or between two different ones).

These observations and suggestions implicitly assume that decision-making is a more or less rational, scientific data driven process. Real-life decision-making is often blurred by institutional and political considerations.

Eriksen et al. (2015) argue that “adaptation is a social-political process that mediates how individuals and collectives deal with multiple and concurrent environmental and social changes” (p. 523). The adaptation pathways approach, like other approaches developed for rationalizing decision-making in the face of uncertainty, does not automatically address the political aspects of decision-making. They argue for “reframing adaptation to take account of how the exercise of power is always present within climate change responses. (…) Our concern is to (…) hold in view how any transformational adaptation pathway will inevitably be plagued by contradictory outcomes” (p. 524).

On the basis of their research, Van Buuren et al. (2016) conclude that “specific mechanisms of path dependency, for example, the existing power asymmetries between competing coalitions and the intricate complexity of flood policies, prevent institutional change, but cannot prevent ideas about resilience slowly gaining more impact.” (p. 41). This implies that conservative powers may block or slow down necessary transformations.

Hermans et al. (2017) have studied the adaptation pathways constructed in the Dutch Delta Programme and conclude that “different types of signposts exist. Technical signposts, in particular, need to be distinguished from political ones, and require different learning processes with different types of actors.”(p. 29). This improves—and complicates—the analysis.

Van der Brugge and Roosjen (2015) point out that the different strategies making up an adaptation map or route map may require different institutional and sociocultural conditions. Climatological and social economic scenarios that favor a certain strategy may not automatically also favor the necessary conditions. They also signal challenges with regard to the governance needed to keep options open for future, an important aspect of the way adaptation pathways were developed in the Dutch Delta Programme. Especially in the case of transfer options that require larger, transitional changes, the institutional and social cultural conditions are important constraint factors (Grin et al. 2010). Van der Brugge and Roosjen (2015) also warn that the resistance to change in the existing social technical regimes should not be underestimated. The governance challenges have remained implicit in adaptation pathway approach—and are considerable.

## Common findings from additional recent experiences of applying the adaptation pathways approach

From a brief literature review of recent experiences of applying adaptation pathways in practice, the following common findings, or “threads”, were distilled.

A *first* common conclusion is that the adaptation pathways approach is *effective in informing and mobilizing decision*-*makers*. Applied in Lakes Entrance, a small community in Eastern Victoria, Australia, local adaptation pathways “create a socially acceptable framework that guides adaptation into the future. Using this approach creates a palatable narrative about adaptation that is time-sensitive, community-sensitive, and owned by local people.” (Barnett et al. 2014, p. 1107). Rosenzweig and Solecki (2014) analyzed the experience in New York with integrating a flexible adaptation pathways approach into the municipality’s climate action strategy and concluded that the concept is useful across cities, since it is size and development-stage neutral. On the basis of experiences in a more rural setting, in Hutt River, located in New Zealand’s lower North Island, Lawrence et al. (2013, p. 133) conclude that “tools to rapidly explore alternative futures can therefore support evaluation of a wider range of response options at exploratory stages of decision-making, which helps avoid planning responses that are predicated on historical experience and a single ‘best estimate’ scenario. This encourages responses to better reflect the changing nature of the risk.” On the basis of case studies in Central Australia, the Brazilian Amazon and the Kalahari in Botswana, Maru and Smith (2014, p. 323) show “how appropriate vulnerability-reducing and resilience-building responses can be combined for (potentially) better outcomes using adaptation pathways.”

A *second* “common thread” through the papers is the *strong call for incorporatinglocal information* in the design of the adaptation pathways, stressing the importance of commitment at the local and regional level. The authors of the EPICCA 2014 study (Siebentritt et al. 2014, p. 3) state that “the regional stakeholders need to be directly involved in the development of a plan if it is to be implemented in a community.” A process of active regional and local engagement is considered crucial. Barnett et al. (2014, p. 1103) conclude “Developing local adaptation pathways that build on triggers of change that have social impacts are salient to local people, which helps build consensus among different constituencies.” Rosenzweig and Solecki (Rosenzweig and Solecki 2014, p. 406) similarly advice to include “co-generation of climate science by scientists and stakeholders”, because they found it to be “an essential element in the development of the flexible adaptation approach in NYC.” This point is also stressed by Smit and Wandel (2006). Engagement of local stakeholders is considered crucial. It contributes to the quality of the plans by incorporating local knowledge and to commitment for the implementation of the plans. It also constitutes the indispensable base for starting a long-term process that will need future adjustments as local conditions continue to evolve (NSOB 2011). Lin et al. (2017) performed an ex-post analysis of eight Australian coastal zone projects on climate adaptation. The results of a project participation survey show that the adaptation pathways were “generally framed narrowly and conservatively to emphasize extant economic, administrative, and legal considerations over community, participatory, or exploratory ones.” It is concluded that adaptive learning requires a co-learning process with stakeholders: it is crucial to organize stakeholder participation in pathways development.

*Thirdly*, it is concluded that *coordination at a higher level* is needed to increase consistency. Among the lessons learned in New York (Rosenzweig and Solecki 2014, p. 406) is “that the flexible adaptation strategies need to be locally appropriate yet regionally coordinated.” In the case of New York, this regional coordination was missing, resulting in a “Balkanization force” that led to large local differences in approaches for rebuilding and resilience (“a patchwork rather than regional fabric for resiliency”).

This point is also made by Kuklicke and Demeritt (2016). In their research, they compare two ways to incorporate climate change in flood safety measures: a “one size fits all” approach used for the river system, prescribing that a 20% increase in peak flood flows should be taken into account, with a more adaptive approach for sea level rise that allows for iterative updates in guidance with the very latest scientific advances. Where the first approach was being regarded by experts as, at best, a makeshift estimate, it proved quite effective in adjusting operational-level plans for fluvial flooding. The scientifically more sophisticated approach used for protection against sea level rise amplified institutional anxieties about whether and how to adapt flood and coastal erosion risk management schemes to climate change and thus worked less well.

A *fourth* element that is distilled is the call for *periodic updates* of the adaptation pathways. Drafting the regional climate change adaptation plan for the Eyre Peninsula, eight sectoral pathways have been developed. Drawing on these pathways, a cross-regional emerging pathway map has been developed. In the spirit of adaptive management, a final action in the plan is to reconsider it periodically, for instance, every 2 to 3 years. (Siebentritt et al. 2014). The concept of flexible adaptation pathways as an approach to responding to climate change was laid out by the New York City Panel on Climate Change in 2010. The NPCC emphasized the flexible adaptation pathways are not fixed; adaptations are defined in terms of acceptable risk levels and are re-evaluated over time, rather than using an approach that sets inflexible standards for adaptation early in the process (Rosenzweig and Solecki 2014)

A *fifth* common point is that the authors agree on the need to *switch*, at some time in the future, *from incremental to transformational strategies*—but that there is little reflection on how this switch can be made. As analyzed by Lonsdale et al. (2015), there is a multitude of options for distinguishing transformational from incremental strategies. For the purpose of this practice-oriented analysis, we have used the following rather straightforward descriptions. Where incremental strategies consist of series of small measures, enough to keep pace with more or less predictable changes in external conditions, transformational strategies are composed of more drastic interventions, altering the system in a more fundamental way, anticipating possible more abrupt and larger changes in external conditions.

“In the short term, many sectors are likely to continue with current best practices that will help to prepare for climate change through incremental change. Within two or three decades, more of the regions adaptation actions will need to focus on protecting assets and starting to transform some sectors. In the long term, adaptation may require retreat and further transformation within sectors. Notably, planning work for many of these medium and long-term action needs to commence now.” (Siebentritt et al. 2014, p. 50)

In this study, adaptation measures are necessarily incremental in the beginning and necessarily transformational in the longer term—but what needs to be done to prepare for this shift is not described.

The research of Barnett et al. (2014, p. 1103) shows that “adaptation pathways are feasible at the local scale, offering a low-risk, low-cost way to begin the long process of adaptation to sea level rise.” The authors seem to focus the application of adaptation pathways on a gradual, smooth start of the adaptation process.

Rosenzweig and Solecki (2014, p. 406) conclude “Prior to hurricane Sandy, incremental adaptation strategies were envisioned so as to avoid disruptions of current systems. (…) After Sandy it has become clear that (…) transformation at full regional scale is required.” Sustaining that transformative trajectory is considered a challenge. “A broad knowledgebase should enable the incorporation of transformative action into flexible adaptation pathways and risk management paradigms throughout the entire region.” So they see possibilities for adaptation pathways to pave the way for transformative interventions.

The same goes for Wise et al. (2014, p. 325) who observe that, although the need to adapt to climate change is now widely recognized, efforts to actually adapt “have not led to substantial rates of implementation of adaptation actions despite substantial investments in adaptation science. Moreover, implemented actions have been mostly incremental and focused on proximate causes; there are far fewer reports of more systematic or transformative actions.”

## Discussion and conclusions

The experience in the UK and the Netherlands with applying adaptation pathways in the TE2100 Project and the Dutch Delta Programme has been compared with the “common findings” from recent literature on other applications of adaptation pathways.

The first common finding from the literature review, that adaptation pathways are effective in informing and mobilizing decision-makers, is in line with our own experiences, as described in Section 5.

The second ‘thread” through the papers, the strong call for incorporating local information in the design of the adaptation pathways and the third point, which stresses the need of coordination at a higher level to ensure consistency, are both recognized from our own experiences. Both in the UK and the Netherlands, these two points have effectively been addressed in the way the programs were structured and processes were organized. In the process of developing the TE 2100 Plan, local parties were actively involved in inventorying and discussing possible measures, while consistency in building up the plan was secured by the coordination from the level of the City of London. In the Netherlands, the structure of the Delta Programme includes both regional sub-programs, in which municipalities, provinces, water boards, NGOs, and the private sector cooperate with the national government in developing regional strategies and the Delta Commissioner for the central coordination of the program.

A fourth element that is distilled from the review is the call for periodic updates of the adaptation pathways. This point was also recognized and effectively addressed in both cases. In the UK, this was done by setting up a monitoring program that will inform a scheduled review and re-appraisal of the TE 2100 Plan every 10 years with a mid-term monitoring review to be undertaken every 5 years. The Dutch Delta Programme has chosen a similar approach: a systematic review of all regional strategies are foreseen every 6 years, and every 12 years, practical experience and research results will be analyzed to determine if the new flood safety standards should be revised.

A fifth common point that arises from the papers is the need to secure the possibility to switch, at some time in the future, from incremental to transformational strategies. The adaptation pathway approach could be improved by making more explicit how such a switch could be facilitated. This point also emerged from the analysis of the two case studies.

The “threads” 1 and 5 overlap with the experience from the two water management programs. The other three common points that emerged from the literature review (threads 2, 3, and 4) focus on the way the construction of adaptation pathways should be organized. They are in line with the way the two water management programs have operationalized that process.

Table 1 summarizes the lessons learned from the analysis.

**Table 1:**
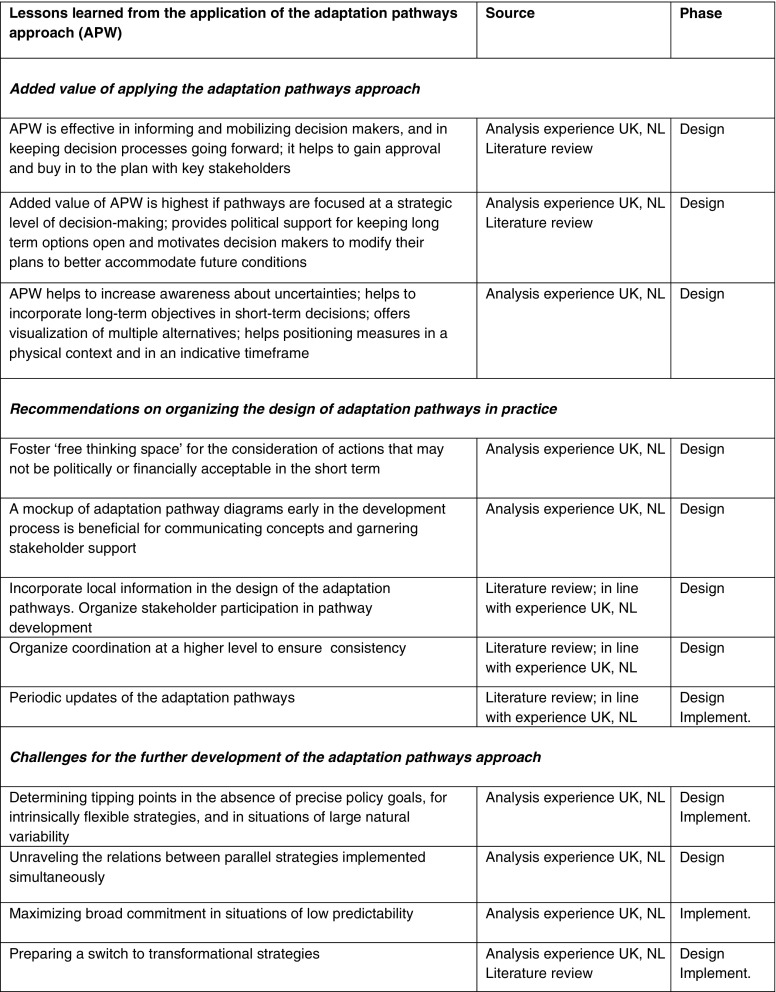
Lessons learned on the application of the adaptation pathways approach

Lin et al. (2017) state that “very little work has been done to evaluate the current use of adaptation pathways and its utility to practitioners and decision makers” (p. 387). By combining the observations from the practical experiences with the results of the literature review, we hope to have made a contribution to filling the observed.

A first conclusion is that the adaptation pathways approach can effectively contribute to pace and quality of decision-making processes that are confronted with large uncertainties about future developments. These positive experiences have led to a broader application of this approach.

The Mayor of London’s infrastructure plan called for the use of adaptation pathways in the development of water resource plans for London (Mayor of London 2015). Thames Water and other water companies in the South East have been investigating their use and demonstration pathways have been developed (Kingsborough et al. 2016). A pathways approach in response to surface water flooding and heat waves is the subject of ongoing research.

In the Netherlands, applications of an adaptive approach are presently being considered in a broad array of policy fields, such as the development of a national strategy for climate mitigation, of a more flexible approach for the national programming of large investments in infrastructural projects, of a national vision on sustainable fuel, of national guidelines for the design and maintenance of tunnels, and of regional strategies for the sustainable use of groundwater reserves.

A second conclusion is that there are still major challenges for the scientific community in the further development of the approach. These notably include the design of a monitoring and evaluation system that is capable of early detection of tipping points in situations with large natural variability. Another specific challenge concerns the preparation of the switch from incremental to transformational strategies. Research is needed on the possibilities of visualizing if, and precluding that, incremental strategies augment path dependency, thereby complicating this switch, that seems unavoidable in the long term.

To effectively deal with the challenges, they need to be taken up in national research programs such as those of the UK Natural Environment Research Council (NERC) and the Dutch National Knowledge and Innovation Programme on Water and Climate (NKWK) and in international research programs such as the EU Joint Programming Initiative on “Water challenges for a changing world.” It equally implies that research results and practical experiences in real life applications need to be discussed in international workshops and seminars. Examples range from large-scale international conferences such as the bi-annual conferences organized by the European Climate Change Adaptation (ECCA) Committee and by the Global Programme of Research on Climate Change Vulnerability, Impacts and Adaptation (PROVIA) to more specialized workshops such as organized by the Society for Decision Making under Deep Uncertainty (DMDU).

## Footnotes

1
https://www.gov.uk/government/publications/thames-estuary-2100-te2100

2 Obviously, the adaptive capacity of a system can be overstretched, as exemplified by species extinctions. When it comes to the question of social limits to adaptation, Adger et al. (2009) state that these are “endogenous to society and hence contingent on ethics, knowledge, attitudes to risk and culture.” (p. 335). The concept of limits to adaptation covers a broader field than floodings and drought. Kingsborough et al. (2017, p. 73) conclude that “without proportionate adaptation, increasing heat-risk is likely to result in increasing heat-related mortality; additional residential overheating; reduced infrastructure performance; and, in extreme cases, it may exceed the threshold for human adaptability and threaten the viability of cities.” Researchers need to begin making progress in predicting and anticipating adaptation limits, and policymakers need to start making plans for managing the consequences of exceeding adaptation limits (Dow et al. 2013, p. 307).
